# “Nomen not est omen”: the (pro)renin receptor and receptor-mediated endocytosis in the proximal tubule—a new (pro)renin-independent role forATP6ap2

**DOI:** 10.1007/s00424-021-02597-0

**Published:** 2021-07-09

**Authors:** Jörg Peters

**Affiliations:** Institute of Physiology, University Medicine of Greifswald, Friedrich-Ludwig-Jahn-Str. 15a, 17475 Greifswald, Germany

Receptor-mediated endocytosis in the kidney is an important mechanism to internalize lipoproteins, hormones, enzymes, or drugs from the tubular lumen. This internalization may be of advantage, if it leads to the recovery of substances which have “escaped” the glomerular filtration barrier and could still be useful. Internalization, however, may be of disadvantage, if nephrotoxic proteins—like in diabetic nephropathy or metabolic syndrome-related nephropathy, are internalized instead of being excreted [1].

Receptor-mediated endocytosis is accomplished by megalin, a fast-recycling receptor that binds and internalizes a variety of ligands. The ligands need to be separated from megalin within the endosomes by v-ATPases. Here the so-called prorenin receptor (ATP6ap2) joins the game.

In the present study, Figueiredo et al. [2] investigated the role of ATP6ap2 in receptor-mediated endocytosis in the proximal tubule. Using two different models, the authors showed that depletion of ATP6ap2 resulted in proteinuria and increased urinary excretion of three megalin ligands. Using transferrin and dextran-FITC as indicators, a specific defect in receptor-mediated endocytosis was confirmed, whereas fluid phase endocytosis remained unaffected.

ATP6ap2 depleted rats, and mice exhibited decreased protein expression of subunits of v-ATPases as well as a mild increase in the number of autophagosomal vacuoles in the kidney. These observations fit well to the known role of ATP6ap2 as an assembly factor for v-ATPases [3] and for a role of ATP6ap2/v-ATPases in autophagy [4, 5]. A change in the activity of the major regulator of autophagy, mTOR, was not observed. However, this finding is not unexpected, since Kissing et al. also discovered mTORC1-independent accumulation of autophagosomes after disruption of ATP6ap2 in hepatocyte [6]. The increase in autophagic vacuoles may represent congestion due to the lack of pH-dependent degradation of the autophagosome content.

The authors were well aware of the known severe developmental defects, and the general organ dysfunctions due to lack of v-ATPase activity disturbed wnt pathways and autophagosome dysfunction (for review, see Peters [7]). Therefore, more specific approaches were chosen to exclude secondary effects as much as possible. First, doxycycline-inducible systems were used in adult rats and mice, allowing regular embryonic and postnatal development prior to ATP6ap2 depletion. Second, the mouse model was specific for kidney epithelial cells, thereby excluding deleterious effects of ATP6ap2 depletion in other cell types including podocytes [4, 5]. Third, severe renal pathology and renal or systemic acidosis could be prevented by using a low-dose doxycycline regime. Finally, in the rat, a shRNA-mediated reduction of ATP6ap2 expression was chosen to avoid a complete deletion (per cell). Despite these precautions, a mild increase in dysfunctional autophagosomes was seen. This may be attributed to the fact that the downregulation of ATP6ap2 was still too strong (90% reduction of ATP6ap2 expression). Nevertheless, taken together, the data allow well the conclusion that ATP6ap2 is essential for receptor-mediated endocytosis in the proximal tubules.

Interestingly, also renin is a ligand of megalin [8]. Thus, although supraphysiological concentrations of (pro)renin are needed to bind the (pro)renin receptor (about 100-fold higher than found even under pathological conditions), these two proteins meet again unexpectedly. The (pro)renin receptor was shown to be involved in the megalin-mediated uptake of renin and other components of the RAS in cell cultures in vitro. [9]. The present study now provides a good argument that this may happen also in vivo, providing another argument for renin uptake as a source for local RAS activity in the kidney.
